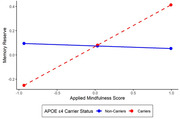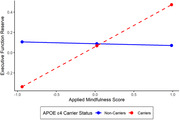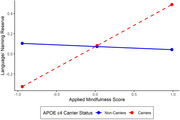# Do associations between mindfulness and cognitive reserve vary by APOE e4 carrier status?

**DOI:** 10.1002/alz.088358

**Published:** 2025-01-03

**Authors:** Deirdre O'Shea, Lilah M Besser, Katana Rader, Andrea S. Zhang, Rebecca Shakour, James E Galvin

**Affiliations:** ^1^ University of Miami Miller School of Medicine, Boca Raton, FL USA

## Abstract

**Background:**

Evidence suggests that mindfulness practice may reduce the risk of dementia by enhancing cognitive reserve. However, it’s unclear whether APOE ε4 carrier status influences the link between mindfulness and cognitive reserve. This study examined whether associations between mindfulness and memory, language, executive and speed reserve differed by APOE ε4 carrier status.

**Method:**

Data from 117 older adults without dementia (age = 67.8+9.8y, 67% female, 30% APOE ε4 carriers) from the Healthy Brain Initiative study were included. Cognitive reserve indices for memory, language, executive function, and speed were the residuals from regressing domain scores onto total hippocampal volume, total gray matter volume, and total white matter hyperintensities volume, with higher residuals indicating more reserve. Mindfulness was assessed using the 15‐item Applied Mindfulness Process Scale (AMPS). APOE ε4 status was coded dichotomously: presence of at least one ε4 allele (1) indicated carrier status, while absence (0) indicated non‐carrier status. Multiple linear regression models were used to test interactions APOE x AMPS on each cognitive reserve domain. All models were adjusted for age, sex, education, and depression levels.

**Result:**

Higher scores on the AMPS were associated with greater memory reserve in APOE ε4 carriers, (β = 0.17, 95% CI [0.01, 0.68]), but not in non‐carriers (β = 0.11, 95% CI [‐0.24, 0.20]). A similar association was found for executive function reserve (ε4 carriers: β = 0.17, 95% [CI ‐0.08 0.76]; ε4 non‐carriers: β = 0.11, 95% CI [‐0.24, 0.20]), and language reserve (ε4 carriers: β = 0.17, 95% CI [0.09, 0.76]; ε4 non‐carriers: β = 0.11, 95% CI [‐0.25, 0.18]). No significant interaction between APOE ε4 status and AMPS was observed for speed reserve.

**Conclusion:**

Mindfulness practice was associated with enhanced cognitive reserve in memory, executive function, and language among APOE ε4 carriers, but not in non‐carriers. This differential impact underscores the potential of mindfulness‐based interventions in improving cognitive outcomes, particularly for those at higher risk for dementia due to APOE ε4 carrier status. The cross‐sectional nature of the study limits the ability to infer causality; however, the patterns observed suggest that future longitudinal studies are warranted to explore these relationships.